# Maltose metabolism in serum free CHO culture involves lysosomal acid α-glucosidase

**DOI:** 10.1038/s41598-025-30901-w

**Published:** 2025-12-04

**Authors:** Tessa Rui Min Tan, Lian Yee Yip, Janice Gek Ling Tan, Dawn Sow Zong Leong, Yan Ni Annie Soh, Shi Ya Mak, Ying Swan Ho, Say Kong Ng

**Affiliations:** 1 Bioprocessing Technology Institute (BTI), Agency for Science, Technology and Research (A*STAR), 20 Biopolis Way, Centros #06-01, 138668 Singapore, Singapore; 2 Food, Chemical and Biotechnology Cluster, Singapore Institute of Technology (SIT), Singapore, Singapore; 3 Singapore Institute of Food and Biotechnology Innovation (SIFBI), Agency for Science, Technology and Research (A*STAR), Singapore, Singapore; 4 Bezos Centre for Sustainable Protein , National University of Singapore, Singapore, Singapore

**Keywords:** Maltose, CHO, Disaccharides, Acid α-glucosidase, Biotechnology, Metabolic engineering, Metabolomics

## Abstract

Mammalian cells have been previously shown to utilize maltose as an energy source for long-term cell growth. To characterize maltose uptake and consumption in CHO-K1, we measured its intracellular concentrations over time and tracked the incorporation of C13-labelled disaccharides into TCA cycle intermediates. Lysosomal acid α-glucosidase (GAA) was identified to potentially be involved in the initial reaction of breaking the α(1 → 4)-glycosidic bond in maltose, releasing two glucose residues for energy metabolism. Using a GAA-KO CHO-K1 cell line, we showed that GAA is involved in CHO-K1 maltose metabolism. Transport of maltose in wild-type and GAA-KO CHO-K1 is concentration-dependent. The rate of maltose uptake for GAA-KO cells was also linear when extracellular maltose is between 0 to 40 g/L, and saturation in uptake kinetics was not observed over that concentration range.

**Supplementary Information:**

The online version contains supplementary material available at 10.1038/s41598-025-30901-w.

## Introduction

Mammalian cell cultures require a carbohydrate source for cell growth and metabolism. The monosaccharide glucose is typically the most important carbohydrate in mammalian cells, and its transport and metabolism have been well studied. Other monosaccharides like fructose and galactose can also be utilized as an energy source for various cell types^1^. Thus far, maltose is the only disaccharide that has been demonstrated to function as an energy source for mammalian cells in protein-free media^2,3^. Unlike other disaccharides like sucrose, lactose or trehalose, maltose can support the growth of CHO and HEK293 mammalian cells in a protein-free medium in long-term cultures. This can be beneficial in mammalian cell-based biopharmaceutical manufacturing, because it can theoretically increase carbohydrate content of the culture medium and decrease lactate production. We have also demonstrated that maltose supplementation improved recombinant monoclonal antibody titer by 15% and 23% in batch and fed batch cultures respectively^3^. By understanding how maltose is transported into cells and metabolized, maltose supplementation in cell culture may be optimized more effectively for cell growth and productivity.

Maltose consists of two glucose residues linked by an α(1 → 4)-glycosidic bond. Various alpha-glucosidases are capable of breaking α(1 → 4)-glycosidic bonds, facilitating the release of glucose from disaccharides and polysaccharides^4^. Maltose may be hydrolyzed by disaccharidases present on the cell membrane, like intestinal maltase-glucoamylase (MGAM) and sucrase-isomaltase (SI)^5^, prior to transport into CHO-K1 cells. However, it is more likely that maltose is internalized by CHO-K1 before being hydrolyzed by intracellular glucosidases (e.g. lysosomal acid alpha-glucosidase (GAA)), as maltose was detected intracellularly through liquid chromatography-mass spectrometry (LC–MS) analysis for host CHO-K1 cells and an antibody-producing CHO-K1 cell line grown in maltose-supplemented media^2^. Maltose was also not broken down into its constituent glucose molecules in conditioned culture media, implying that secreted disaccharidases are unlikely to be implicated in maltose metabolism in CHO-K1 cells^2^. Identifying the enzyme involved in the breakdown of maltose into its constituent glucose molecules may potentially facilitate the identification of other cell lines where maltose supplementation may have beneficial effects. Additionally, it may open further avenues in using cell line engineering to possibly enhance the effect of maltose supplementation.

In this report, we characterized the metabolism of disaccharides in CHO-K1 cells by quantifying intracellular disaccharide levels to determine their uptake and consumption profiles. The incorporation of labeled maltose into various TCA cycle intermediates was also measured. GAA was also identified as being involved in the conversion of internalized maltose to glucose, before being used as an energy source for cellular metabolism. Maltose uptake kinetics were calculated for GAA-KO and non-KO cells in media with increasing maltose concentrations, to identify the internalization mechanism of maltose in CHO-K1.

## Material and methods

### Cell lines and cell cultivation

Suspension adapted CHO-K1 cell line (ATCC, Manassas, VA) was cultured in an in-house DMEM/F12-based protein free chemically defined medium (PFCDM) supplemented with 6 g/L glucose (Sigma-Aldrich, St. Louis, MO), 8 mM L-glutamine (Sigma-Aldrich) and 0.1% Pluronic® F-68 (Thermofisher, Waltham, MA). Cells were routinely passaged every 3 to 4 days in 125 mL single-use disposable Erlenmeyer flasks (Corning, Corning, NY), and maintained in a humidified 37 °C/8% CO_2_ incubator with a shaker platform set to 110 rpm. Cell densities and viabilities were determined using the Vi-CELL XR Cell Viability Analyzer (Beckman Coulter, Brea, CA), according to manufacturer’s instructions.

### Glucosidase inhibitor testing on parental and maltose-adapted CHO-K1

Wild-type and maltose-adapted CHO-K1 cells were cultured in a protein-free medium, HyQ PF-CHO MPS (Cytiva, Marlborough, MA) supplemented with either 3.6 g/L glucose (Sigma-Aldrich) or maltose (Sigma-Aldrich), 6 mM L-glutamine (Sigma-Aldrich) and 0.1% Pluronic® F-68 (Thermofisher). Castanospermine and Acarbose were dissolved in DMSO (Sigma-Aldrich) or water respectively, before being added to the culture medium at various concentrations. Cells were cultivated at a seeding density of 4 × 10^5^ cells/mL in disposable Erlenmeyer flasks (Corning) and their viable cell densities and cell viabilities monitored daily using the Vi-CELL XR Cell Viability Analyzer (Beckman Coulter), according to manufacturer’s instructions.

### Shake flask batch cultures sampling

For growth comparison of CHO-K1 cells in PFCDM with glucose and maltose as carbohydrate sources, CHO-K1 cells were seeded into glucose-containing PFCDM supplemented with L-glutamine (Sigma-Aldrich) and 0.1% Pluronic® F-68 (Thermofisher). Disaccharides, in the form of maltose (Sigma-Aldrich) and trehalose (Sigma-Aldrich), or UL-13C12 labelled maltose and trehalose (Omicron Biochemicals, South Bend, IN) were also added to the culture medium. Cells were cultivated in single-use Erlenmeyer flasks (Corning), at specified seeding densities in single-use Erlenmeyer flasks (Corning). For biochemical analysis of cell culture supernatant, 1 mL of cell culture supernatant was centrifuged at 8000 rpm for 10 min, and the clarified supernatant analyzed using a Bioprofile 100 Plus analyzer (Nova Biomedical, Waltham, MA) to obtain their respective extracellular glucose, lactate, glutamine and ammonium concentrations.

### Growth of acid alpha glucosidase (GAA)-knockout (KO) CHO-K1

GAA-KO CHO-K1 cells and a null-KO control were obtained from Genscript and initially grown in suspension cultures with DMEM medium (Thermofisher) supplemented with 5% fetal bovine serum (FBS) (Cytiva) and subsequently adapted in a stepwise manner to grow in commercial glucose-containing CD OptiCHO medium (Thermofisher). Cells were routinely subcultured every 3 – 4 days, using the same cultivation procedures as parental CHO-K1.

### Verification of GAA expression in CHO-K1

After adaption to growth in serum-free CD OptiCHO medium, RNA was extracted using RNeasy Mini Kit (Qiagen, Hilden, Germany) from GAA-KO and null-KO cells and quantified using a Nanodrop 2000 spectrophotometer (Thermofisher). A OneStep RT-PCR kit (Qiagen) was then used for reverse transcription and amplification of cDNA using sequence-specific primers. PCR products were extracted from agarose gels using NucleoSpin Gel and PCR Clean-up Kit (Machery Nagel, Düren, Germany) and sequenced.

To probe for intracellular GAA protein expression, cells were harvested and lysed using CellLytic M Mammalian Cell Lysis Reagent (Sigma-Aldrich) supplemented with Protease Inhibitor Cocktail (Nacalai Tesque, Kyoto, Japan). Cell lysate samples were analyzed via SDS-PAGE and Western blotting using 4–12% NuPage Bis–Tris gels (Thermofisher). The PVDF membrane was then probed using antibodies against GAA (ab137068, Abcam, Cambridge, UK) and actin (A2066, Sigma Aldrich).

### Overexpression of GAA

CHO GAA coding sequence (NCBI reference sequence XM_007647236 verified by RT-PCR from cDNA of the parental CHO-K1 cell line) (Genscript, Piscataway, NJ) was cloned into an in-house dicistronic IRES mammalian expression vector backbone containing a Zeocin resistance marker. Expression vectors were purified with Nucleobond Xtra Midi EF (Machery Nagel) according to manufacturer’s instructions. Plasmid DNA quality and concentration was analyzed using the Nanodrop 2000 spectrophotometer (Thermofisher). Plasmids were linearized with BstBI (New England Biolabs, Ipswich, MA), purified using sodium acetate and ethanol precipitation and resuspended in sterile nuclease-free water (1^st^ Base, Singapore). Using the Amaxa 4D Nucleofector (Lonza, Basel, Switzerland) with SG solution and pulse code FF-137, 1 × 10^7^ cells from a 48 h culture were transfected with 4 µg of linearized plasmid according to manufacturer’s instructions and incubated in 6-well microplates in a static incubator. At 48 h post-transfection, cells were grown DMEM/F12 supplemented with 5% FBS and 600 µg/mL Zeocin (Thermofisher) in static 6-well microplates and sequentially adapted to grow in OptiCHO (Thermofisher) with 600 µg/mL Zeocin in suspension culture. Experiments were performed on stably transfected cells after culture viability was consistently above 90%.

### Extraction of intracellular metabolites and detection of intracellular maltose

1 × 10^7^ cells in media are first quenched via the addition of five volumes of ice-cold 150 mM NaCl (Sigma-Aldrich) and centrifugation at 4500 rpm at 4 °C, for 4 min. After aspiration of the supernatant, the process was repeated with 10 mL of 150 mM NaCl solution, to obtain the final cell pellet used for intracellular metabolite extraction. 10 µL of a 0.4 mM UL-13C12 maltose (Omicron) solution was added as a reference standard. A two-phase liquid extraction protocol^6^, using methanol (Merck Millipore, Burlington, MA), chloroform (Merck Millipore) and 3.8 mM Tricine (Sigma-Aldrich) (in 9:10 proportion with methanol) was used to extract intracellular disaccharides, and the resulting extracts stored at -80 °C. Aqueous cell extracts were dried under vacuum at 4 °C using a Centrivap (Labconco, Kansas City, MO) and reconstituted in methanol/0.1% NH_3_, prior to analysis using Acquity UPLC-QDa (Waters Corporation, Milford, MA). Mobile phases of water and acetonitrile (Optima grade, Fisher Scientific, Pittsburg, PA) with 0.1% NH3, at a flow rate of 0.4 ml/min, were used for gradient separation through a BEH amide 1.7 µm 2.1 × 100 mm column (Waters) at 60 °C. Peak areas of disaccharides were integrated using Empower software (Waters), and compared against the peak area of the UL-13C12 maltose reference standard. While relative peak area analysis is less accurate than absolute quantification, the response factors of C12 and C13 maltose are relatively similar and using a C13 maltose reference standard can account for potential losses during the metabolite extraction process.

### Quantification of intracellular disaccharides

Standard curves for unlabelled and labelled maltose and trehalose, in CHO-K1 intracellular extract, were obtained through UPLC-QDa analysis. Relative response factors were calculated for the unlabelled disaccharide and its UL-13C12 labelled counterpart. Intracellular disaccharide concentrations were calculated based on the equivalent number of cells in the analyzed extract, using average cell diameters measured using the Vi-CELL XR Cell Viability Analyzer (Beckman Coulter).

### Measuring the incorporation of ^13^C into intracellular downstream metabolites by mass spectrometry and isotopic carbon tracing

For tracing of the uptake/consumption of disaccharides by CHO-K1 cells, the cells were cultured as described in the methods sub-section “Shake flask batch culture sampling”, with the addition of 10 g/L of ^13^C-labelled maltose or trehalose to the PFCD culture media. 1 × 10^7^ cells were harvested for metabolite extraction at 0 h, 0.5 h, 48 h, and 96 h. Intracellular metabolites were subsequently extracted using two-phase liquid–liquid extraction protocol^6^ (extraction solvents include methanol (Optima grade, Fisher Scientific), chloroform (J.T. Baker, Center Valley, PA), and 3.8 mM tricine (Sigma–Aldrich):methanol (9:10) mixture), dried, and stored at -80 °C. The dried samples were reconstituted in 95:5 (v/v) water:methanol prior to analysis. The polar metabolites were analyzed by LC–MS using an ACQUITY UPLC system (Waters Corporation, Milford, MA) in tandem with a mass spectrometer (QExactive™, Thermofisher). The LC method^7^ was based on the use of a reversed phase column with polar encapping (ACQUITY UPLC HSS T3, Waters). Electrospray ionization (ESI) was conducted in both positive and negative modes with a mass range of 70 to 1050 m/z at a resolution of 70,000. Sheath and auxiliary gas flow were set at 30.0 and 20.0 (arbitrary units) respectively, while a capillary temperature of 400 °C was used. The spray voltage was 1.5 kV and 1.25 kV respectively, for both ionization modes.

Processing of raw data and peak matching to the predicted m/z of unlabelled and ^13^C-labelled TCA cycle metabolites was performed using TraceFinder™ v3.3 (Thermofisher). The mass isotopomer distributions were corrected for natural isotope abundance using IsoCor^8^.

## Results

### Growth and intracellular disaccharide profiles of CHO-K1 cell culture supplemented with maltose or trehalose

We have previously demonstrated that maltose can support the growth of CHO-K1 cells in the absence of glucose, unlike other disaccharides such as trehalose, sucrose and lactose^2^. As these cells grow much slower in glucose-free maltose-supplemented medium, practical application of maltose was demonstrated in glucose-containing maltose supplemented culture, where maltose metabolism continued to support cell growth when glucose was depleted^2,3^. To elucidate the mechanisms of maltose metabolism, we first investigated its transport and accumulation into the cells. Trehalose was used for comparison since it cannot support cell growth and is thus likely not metabolized. Cells were seeded at 0.35 × 10^6^ cells/mL into in-house PFCDM containing 4 g/L glucose, with 10 g/L of additional maltose or trehalose. Viable cell densities and culture viabilities were measured at 0, 0.5, 48 and 96 h, and intracellular metabolites were extracted from 1 × 10^7^ cells at these time-points.

**Fig. 1:**
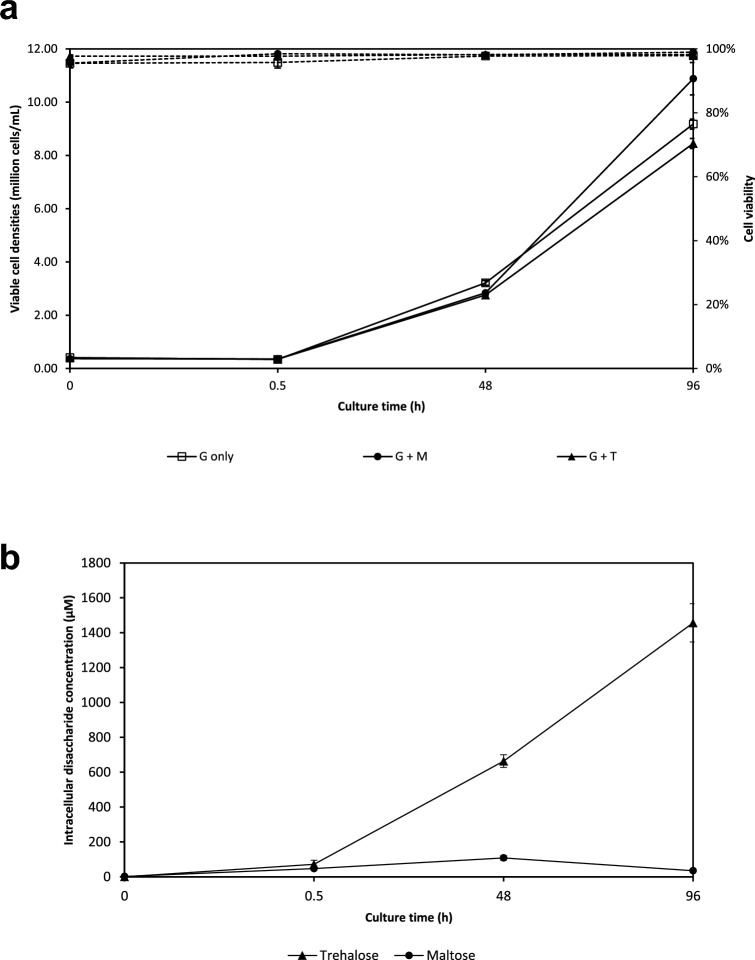
Growth profiles of CHO-K1 cells cultivated in protein-free chemically defined medium (PFCDM) with glucose, supplemented with maltose or trehalose, and quantification of intracellular disaccharide accumulation. CHO-K1 cells originally cultured in PFCDM with 6 g/l of glucose were sub-cultivated in PFCDM with 4 g/l glucose (□) and either 10 g/l of C12 maltose (●) or trehalose (▲). The cultures were monitored at 0, 0.5, 48 and 96 h post-inoculation to obtain their  (a) viable cell densities (────) and cell viabilities (┅┅┅┅ ) and (**b**) intracellular disaccharide concentrations. Mean and s.d. values from 2 independent replicates are plotted.

At 96 h, CHO-K1 cells grown with 10 g/L of maltose supplement achieved higher viable cell densities of over 1 × 10^7^ cells/mL, while cells grown with 10 g/L of trehalose supplement had lower viable cell densities of around 8.5 × 10^6^ cells/mL (Fig. 1a). CHO-K1 grown with 4 g/L glucose without any additional disaccharide supplementation grew to higher cell densities compared to cells grown with trehalose, and lower cell densities compared to cultures containing maltose, suggesting that trehalose is unlikely to be utilized for CHO-K1 growth and energy metabolism. Culture viabilities were higher than 95% throughout the culture duration and were similar across all samples. Glucose concentrations were below 0.2 g/L and relatively similar for all samples at 96 h, implying that upon glucose depletion in the culture medium, maltose metabolism can support CHO-K1 growth to higher cell densities compared to cultures grown without maltose, which corroborates with previous reports^2,3^.

Disaccharide concentrations in the metabolite extracts were determined using UPLC-QDa analysis. Peak intracellular trehalose concentrations of around 1400 µM were observed at 96 h, which was considerably higher than the corresponding 96 h maltose concentration of 35 µM (Fig. 1b). Trehalose concentrations also increased consistently over time, while maltose levels peaked at 48 h at 108 µM and were lower at 96 h. Both maltose and trehalose are detected intracellularly at 0.5 h post-inoculation, indicating that the transport of maltose and trehalose into CHO-K1 can take place within a relatively short time frame. Intracellular maltose and trehalose accumulation was not observed in the 0 h samples, implying that cells cultured in glucose-only medium do not produce or accumulate maltose or trehalose if the culture medium is not supplemented with disaccharides.

These observations of intracellular disaccharide profiles are surprising because it is generally accepted that disaccharides are polar molecules that are too large to pass through the cell lipid bilayer membrane, and few animal disaccharide transporters have been reported thus far^9,10^. Nonetheless, these profiles corroborate with previous observations: Intracellular trehalose is observed to increase with time, suggesting that it is accumulated intracellularly but not metabolized. On the other hand, intracellular maltose peaked at 48 h and then decreased, suggesting transport into the cells and metabolism with glucose depletion.

### Tracing ^13^C-maltose and trehalose utilization in wild-type CHO-K1 cells

**Fig. 2:**
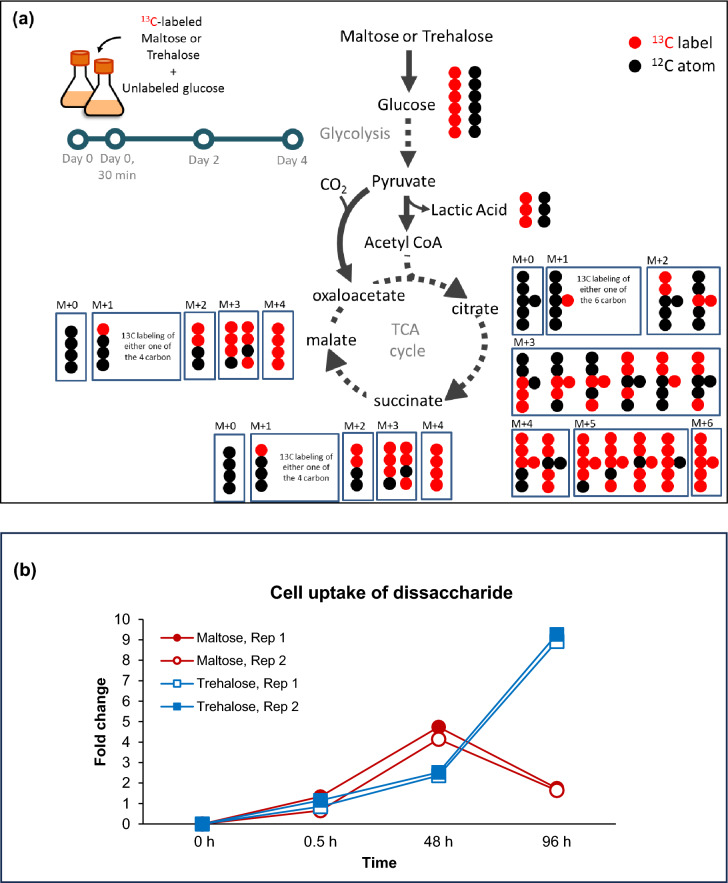

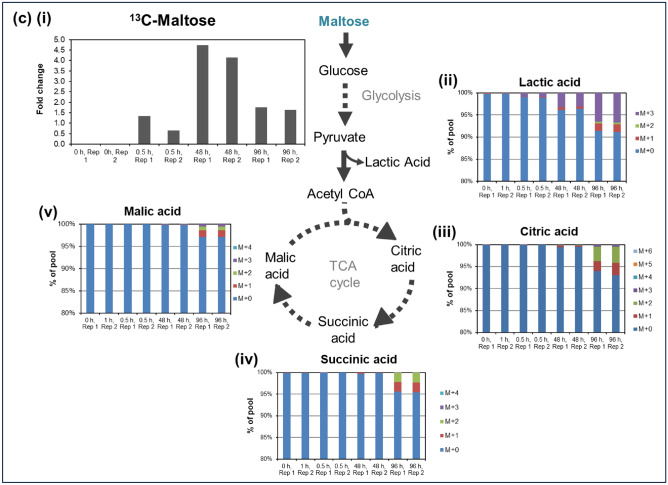

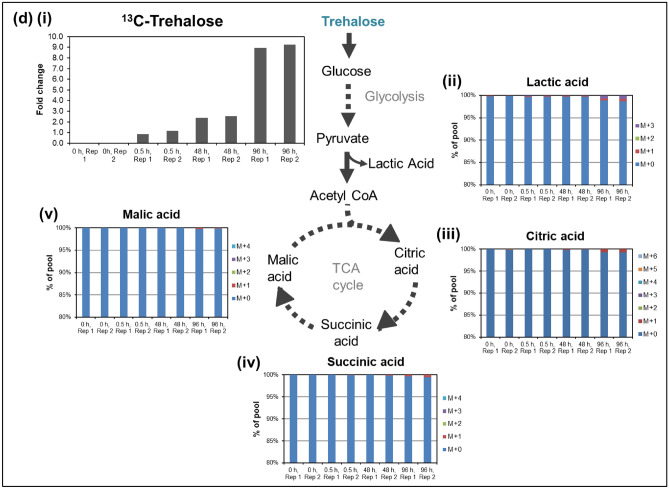
Tracking and distribution of labelled TCA intermediates from CHO-K1 cells grown in glucose-containing medium containing C13-labelled maltose or trehalose (**a**) Schematic of [U-^13^C]maltose and [U-^13^C]trehalose metabolism along the TCA cycle in wild-type CHO-K1 cultured with unlabeled glucose. All possible permutations of ^13^C-labelled intermediates are shown, with the labelled carbons denoted as red dots. The unlabeled glucose was added to the culture media at the start of the experiment; the fully ^13^C-labelled glucose is derived from the breakdown of ^13^C-maltose or ^13^C-trehalose in the culture media. (**b**) Cell uptake of maltose and trehalose over time (with reference to 30-min time point). (**c**) Maltose and (**d**) Trehalose metabolism in CHO-K1 cells – (i) concentration (by peak area) of intracellular maltose detected in CHO-K1, and mass isotopomer analysis of (ii) lactic acid, (iii) citric acid (citrate), (iv) succinic acid (succinate) and (v) malic acid (malate) in cells cultured with media containing ^13^C-maltose or ^13^C-trehalose, and unlabeled glucose.

To determine whether unlabelled and labelled maltose or trehalose act similarly on CHO-K1, growth and biochemical profiles of CHO-K1 grown in 4 g/L glucose and 10 g/L unlabelled or UL-13C12 trehalose or maltose were compared. A seeding density of 0.35 × 10^6^ cells/mL was used. At 0, 0.5, 48 and 96 h, viable cell densities and culture viabilities were measured. Biochemical analysis was also performed on the culture supernatant. Both unlabelled and labelled disaccharide cultures had similar growth and biochemical profiles at the selected points, indicating that both unlabelled and labelled maltose and trehalose have similar biological effects on CHO-K1(Supplementary Figures S1, S2). To further investigate the utilization pathway of maltose by CHO-K1 cells, isotope-labelling experiments were carried out. Cells were cultured in 4 g/l glucose-containing medium supplemented with ^13^C-maltose- or ^13^C-trehalose for 96 h and cell samples obtained at 0 h, 0.5 h, 48 h and 96 h (n = 2), to track the ^13^C enrichment in TCA cycle intermediates by LC–MS (Fig. 2a).

Six units of ^13^C from labelled-glucose, broken down from the labelled disaccharide, undergo glycolysis to form either 3-carbon lactic acid, or 2-carbon acetyl-CoA that enters the TCA cycle (Fig. 2a). The appearance of ^13^C-labelled TCA cycle intermediates begins with the [M + 2] citrate isotopomers, which arise from the first cycle. Successive cycles and incorporation of other carbon sources resulted in partial labelling and the appearance of other isotopologues.

As shown in Fig. 2b, the uptake of both labelled disaccharides into the cells was observed at 0.5 h after the media containing ^13^C-labelled maltose or ^13^C-labelled trehalose was introduced. While the levels of intracellular ^13^C-trehalose increased between 0.5 h to 96 h, intracellular ^13^C-maltose levels saw a peak at 48 h and subsequently decreased at 96 h. These trends are similar to our previous experiment on the addition of unlabelled maltose or trehalose to CHO-K1 cells (Fig. 1).

Figure 2ci–v and 2di–v first show the fold change of ^13^C-maltose and ^13^C-trehalose levels respectively in CHO-K1 cells over 96 h, followed by the percentage of isotopologues incorporation in comparison to the total abundance of lactic acid and TCA cycle intermediates identified from post-processing of LC–MS data (after natural abundance correction) for ^13^C-labelled maltose and ^13^C-labelled trehalose respectively. In Fig. 2cii, the appearance of ^13^C-labelled lactic acid from 0.5 h and TCA cycle intermediates from 48 h in the CHO-K1 cell culture supplemented with ^13^C-labelled maltose indicates that the cells were able to utilize maltose as a source of energy. Specifically, the proportion of ^13^C-labelled lactic acid [M + 3] increased from approximately 0.85% at 0.5 h to 3.2% at 48 h and 6.6% at 96 h. Similarly, the isotopologues of TCA cycle intermediates citrate, malate and succinate (Fig. 2ciii-v) in the ^13^C-maltose fed reactor were detected at 48 h (less than 1% of total abundance) and showed a 1%—3% increase in total abundance between 48 and 96 h.

In contrast, cells supplemented with ^13^C-trehalose showed minimal incorporation of labelled lactic acid and TCA cycle intermediates only at 96 h, suggesting that trehalose was not as readily utilized as an energy source. Specifically, ^13^C-labelled lactic acid [M + 3] only comprised of 0.8% of total lactic acid at 96 h (Fig. 2cii). Similarly, total ^13^C-labelled species of TCA intermediates in these cultures only made up approximately 1.5% of the total abundance (Fig. 2iii-v).

When cultured in glucose-containing media, trehalose involvement in CHO-K1 energy metabolism in the TCA cycle is lower than that of maltose. It is worth noting that while incorporation of ^13^C into TCA cycle intermediates is higher for cells supplemented with ^13^C-maltose compared to ^13^C-trehalose, the overall incorporation of ^13^C into TCA cycle intermediates is relatively modest for both labelled disaccharides. Since maltose may require an additional hydrolysis step to break it down into its constituent glucose molecules before its involvement in energy metabolism, glucose present in the culture medium may be utilized preferentially. In addition, the ^13^C derived from maltose may also be involved in other metabolic pathways.

There is an increase in the proportion of labelled lactic acid and TCA cycle intermediates in the ^13^C-maltose fed system at an earlier time point, corresponding to the lower intracellular ^13^C-maltose levels. The decrease in intracellular ^13^C-maltose between 48 and 96 h indicates that the cells more readily utilize maltose as a carbon source (Fig. 2ci). In contrast, the intracellular ^13^C-trehalose levels increased over the period of 96 h with less than 1% of labelled lactic acid and TCA cycle intermediates shows uptake but minimal utilization of trehalose by the cells. (Fig. 2di).

Although both maltose and trehalose consist of two units of glucose, the glucose subunits in trehalose are linked by an α(1 → 1) glycosidic bond, while the glucose subunits in maltose are linked by an α(1 → 4)-glycosidic bond. Thus far, maltose is the only disaccharide that has been demonstrated to function as an energy source for mammalian cells (CHO and HEK293) in protein-free media to date^2^.

### Identification of enzyme involved in maltose metabolism using various glucosidase inhibitors

**Fig. 3:**
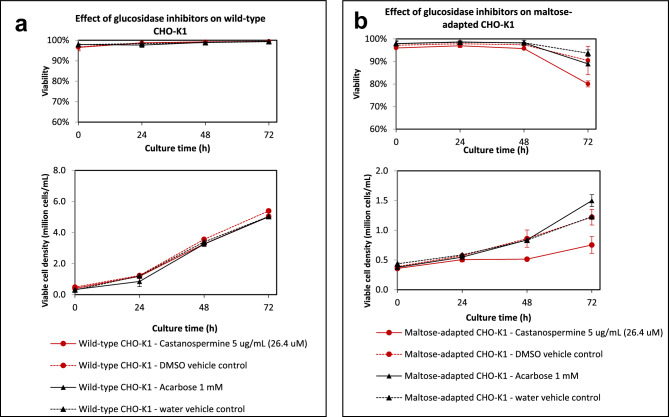
Effect of glucosidase inhibitors on wild-type and maltose-adapted CHO-K1. Wild-type and maltose-adapted CHO-K1 were cultured in protein-free HyQ PF-CHO MPS medium in the presence of glucosidase inhibitors Castanospermine and Acarbose ((────), and their respective vehicle controls (┅┅┅┅). Viable cell densities and cell viabilities were measured. Either glucose or maltose was the only carbohydrate source for wild-type and maltose-adapted CHO-K1 respectively. (**a**) Both inhibitors did not have an inhibitory effect on the growth of wild-type CHO-K1. Acarbose, a glucosidase inhibitor that cannot pass through the cell membrane, did not inhibit the growth of maltose-adapted CHO-K1. (**b**) Viable cell densities of maltose-adapted CHO-K1 supplemented with Castanospermine, a glucosidase inhibitor that can enter the cell, were significantly lower. Mean and s.d. values from 2 independent replicates are plotted.

Wild-type and maltose-adapted CHO-K1^2^ were cultured in protein-free HyQ PF-CHO MPS medium in the presence of glucosidase inhibitors, with either glucose or maltose as the only carbohydrate source respectively. Glucosidase inhibitors act via inhibiting the breakdown of carbohydrates, potentially inhibiting the breakdown of maltose into its two glucose subunits. Both acarbose and castanospermine are competitive inhibitors of glucosidase; however, unlike acarbose^11^, castanospermine is able to pass through the cell membrane and enter cells^12^. Glucose is important for various cell metabolic processes, and low glucose concentrations will negatively affect cell growth. At concentrations of 26.4 µM (5 µg/mL) castanospermine or 1 mM acarbose, both compounds did not exert an inhibitory effect on the growth of wild-type CHO-K1 grown in glucose-containing medium cultured over a 72 h period (Fig. 3a). Similar viable cell densities and cell viabilities were observed in either the presence of these glucosidase inhibitors and their respective vehicle controls, indicating that they are not toxic to CHO-K1 at these concentrations in glucose-containing medium. For maltose-adapted CHO-K1 grown in media containing maltose as the only carbohydrate source, 26.4 µM (5 µg/mL) castanospermine inhibited cell growth compared to its DMSO vehicle control, with viable cell densities of 0.75 × 10^6^ cells/mL achieved at 72 h, compared to 1.2 × 10^6^ cells/mL for the vehicle control (Fig. 3b). Cell viabilities at 72 h were also around 80%, which was lower than the 94% viability observed for its vehicle control. In contrast, addition of 1 mM acarbose did not affect the growth of maltose-adapted CHO-K1 compared to its water vehicle control. Reduced growth of maltose-adapted CHO-K1 cell growth in the presence of castanospermine, but not acarbose, suggests that the enzyme involved in CHO-K1 maltose metabolism is likely to be present intracellularly in CHO-K1 cells. This also suggests that the maltose molecule is transported into CHO-K1 before being broken down into its constituent two glucose subunits for subsequent energy generation, which is in line with the observations in a previous report^2^ and corroborates with results shown in Figs. 1 and 2. As castanospermine is known to be an effective inhibitor of human lysosomal alpha-glucosidase, but not other lysosomal glucosidases^13^, we next investigated the involvement of this glucosidase in maltose metabolism.

### Growth and biochemical profiles of CHO-K1 GAA-KO cells

Lysosomal acid alpha glucosidase (GAA) is an enzyme that is involved in the breakdown of glycogen in human lysosomes^14^, and is hypothesized to be implicated in CHO-K1 maltose metabolism. A GAA-KO cell line and one null knockout control, obtained from Genscript, were adapted to grow in serum-free CD OptiCHO medium containing glucose. Through genomic DNA sequencing, the GAA-KO cell line had 10 bp deleted on one allele and 1 bp inserted on the other allele at the targeted site. Knocking out GAA does not appear to be lethal to CHO-K1 cells, and the cells were eventually adapted to grow in serum-free glucose-containing OptiCHO. RT-PCR was subsequently performed on the GAA-KO cell lines to confirm the disruption of the GAA gene.

**Fig. 4:**
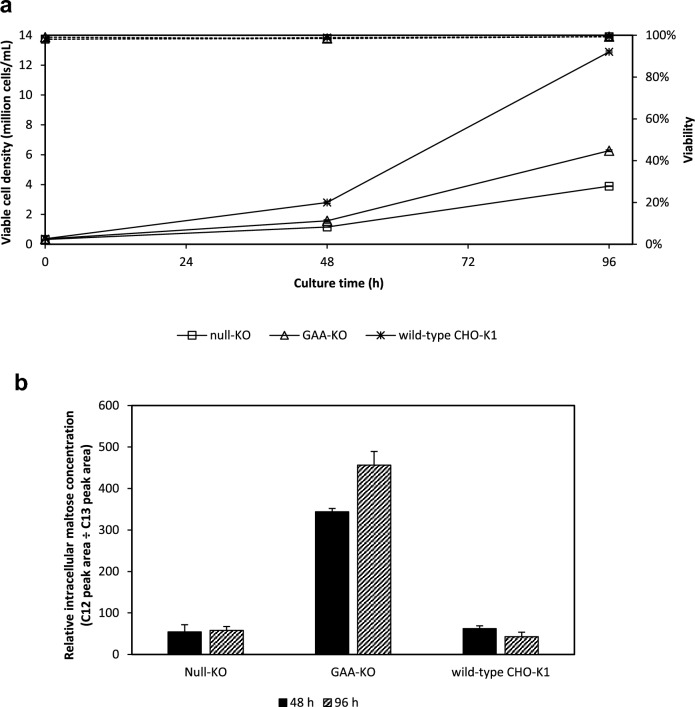
Growth and metabolite profile of GAA-KO, Null-KO and wild-type CHO-K1 cells. CHO-K1 cells originally cultivated in glucose-containing CD OptiCHO were subsequently grown in OptiCHO supplemented with 10 g/l of maltose. The cultures were monitored at 0, 48 and 96 h post-inoculation to obtain their (**a**) viable cell densities (────) and cell viabilities (┅┅┅┅) and (**b**) relative intracellular maltose concentrations at 48 and 96 h. Mean and s.d. values from 2 independent replicates are plotted. Wild-type CHO-K1 cells have been adapted over prolonged periods to serum-free medium, which may account for its significantly better growth compared to null-KO CHO-K1 cells. Relative intracellular maltose are around 5-times higher for GAA-KO cells, compared to null-KO and wild-type CHO-K1 cells.

Wild-type CHO-K1, adapted GAA-KO and adapted null-KO cells were grown in glucose-containing OptiCHO supplemented with 10 g/L maltose, and their viable cell densities and culture viabilities monitored at 0, 48 and 96 h, with 1 × 10^7^ cells collected for intracellular metabolite extraction at these time points. Viable cell densities for the null-KO and GAA-KO cells were similar while wild-type CHO-K1 cells showed much higher viable cell densities, possibly due to prolonged culture in serum-free medium (Fig. 4a).

Relative intracellular maltose concentrations were then measured with 10 µL of a 0.4 mM UL-13C12 maltose solution added during the initial metabolite extraction process as a reference standard. Relative levels of intracellular maltose were similar between wild-type CHO-K1 and null-KO cells, despite differences in their growth curves. Compared to wild-type CHO-K1 and the null-KO cells, relative levels of intracellular maltose in GAA-KO cells at 48 h were 3 to 6-times higher and this increases further at 96 h (Fig. 4b). This trend is similar to that of trehalose shown in Figs. 1b and 2b, suggesting that maltose metabolism is impeded for the GAA-KO cell line, and that maltose metabolism in CHO-K1 is likely to be inhibited when the GAA gene is not expressed.

### Overexpression of GAA in GAA-KO CHO-K1

**Fig. 5:**
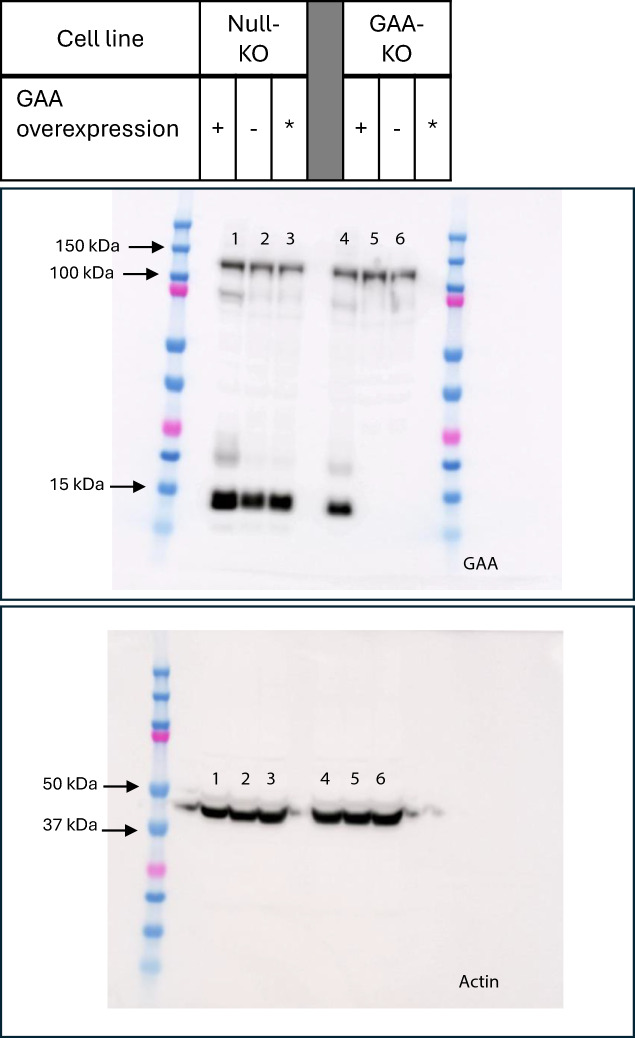
Verification of GAA protein expression via Western blotting. Cell lysate was harvested from GAA-KO and null-KO cells transiently transfected with ( +) GAA or (-) null plasmids, and subsequently analysed using Western blotting. * Denotes untransfected cells. GAA-KO cells have protein sequences disrupted from approximately AA350. The primary antibody (Abcam ab137068) used recognizes the human GAA sequence between AA170 and AA200, which has about 80% sequence homology with CHO GAA. Bands detected below 15 kDa were observed for the null-KO and for cells overexpressing GAA, but not for the null-transfected and untransfected GAA-KO cells. The band above 100 kDa was present for all samples regardless of whether GAA is knocked out or overexpressed. Protein loading was determined via actin detection at around 42 kDa on a separate gel loaded with the same samples and processed in parallel, and was similar across all samples.

To confirm the effect of GAA gene expression on CHO-K1 cells, GAA-KO cells were either transfected with GAA to rescue GAA expression, or with a null plasmid. GAA protein expression was verified via Western blotting, from the extracted cell lysate of transiently transfected cells (Fig. 5). For GAA-KO cells, the GAA protein sequence was disrupted from approximately AA350. The primary antibody (Abcam ab137068) recognizes an immunogen sequence of between AA170 and AA200 of human GAA, which has about 80% sequence homology with CHO GAA. GAA consists of four polypeptides^15^, and the band detected at below 15 kDa may represent a polypeptide containing a disrupted amino acid sequence after AA350. Bands slightly below 15 kDa were observed for the null-KO and for cells overexpressing GAA, but not for the null-transfected and untransfected GAA-KO cells. A band above 100 kDa, observed for all samples regardless of GAA-KO or overexpression, may consist of a polypeptide including the GAA sequence between AA170 and AA200. Using actin as a loading control, protein loading was observed to be relatively similar across all samples tested.

**Fig. 6:**
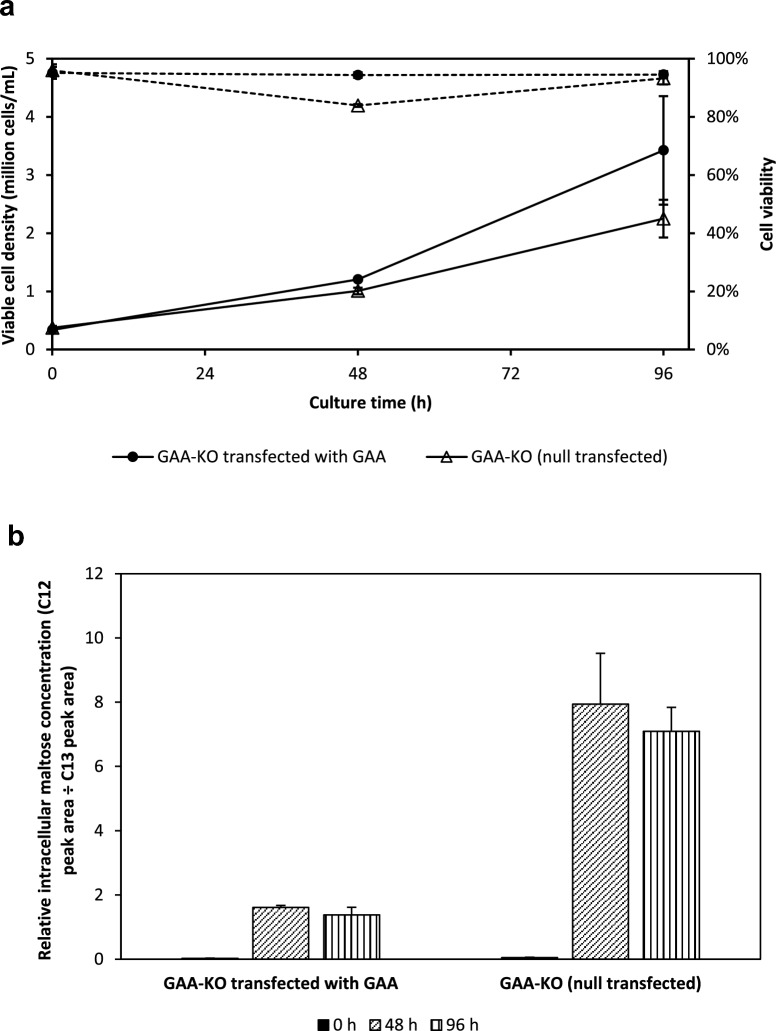
Overexpression of GAA in GAA-KO CHO-K1: growth and metabolite profiles of stably transfected GAA-KO CHO-K1 cells. CHO-K1 cells originally cultivated in glucose-containing OptiCHO were subsequently grown in OptiCHO with 10 g/l of maltose. The cultures were monitored at 0, 48 and 96 h post-inoculation to obtain their (**a**) viable cell densities (────) and cell viabilities (┅┅┅┅) and (**b**) relative intracellular maltose concentrations at 48 and 96 h. Mean and standard deviations values from 2 independent replicates are plotted.

Following selection and stable growth in glucose-containing CD OptiCHO (Thermofisher) with 600 µg/mL Zeocin (Thermofisher), cells were cultured in OptiCHO supplemented with 10 g/L maltose. Cell growth and viabilities were measured at 0, 48 and 96 h, with intracellular metabolites also extracted from 1 × 10^7^ cells on these times. GAA-KO cells stably transfected with the GAA gene had higher viable cell densities of 3.4 × 10^6^ cells/mL at 96 h than GAA-KO cells transfected with the null plasmid (2.3 × 10^6^ cells/mL) (Fig. 6a). Even with similar cell growth at 48 h, GAA-KO cells overexpressing the GAA gene also had relatively lower intracellular maltose levels compared to null-transfected GAA-KO cells (Fig. 6b). Compared to null-transfected cells with a C13-relative peak area of 7.9, rescued GAA-KO cells had a lower relative peak area of 1.6, implying that rescued GAA-KO cells are now able to metabolize maltose intracellularly. Day 4 intracellular maltose levels are also higher for the null-transfected cells compared to the rescued cells, suggesting that GAA is involved in CHO-K1 maltose metabolism.

### Transport of maltose into CHO-K1 cells at varying extracellular maltose concentrations

**Fig. 7:**
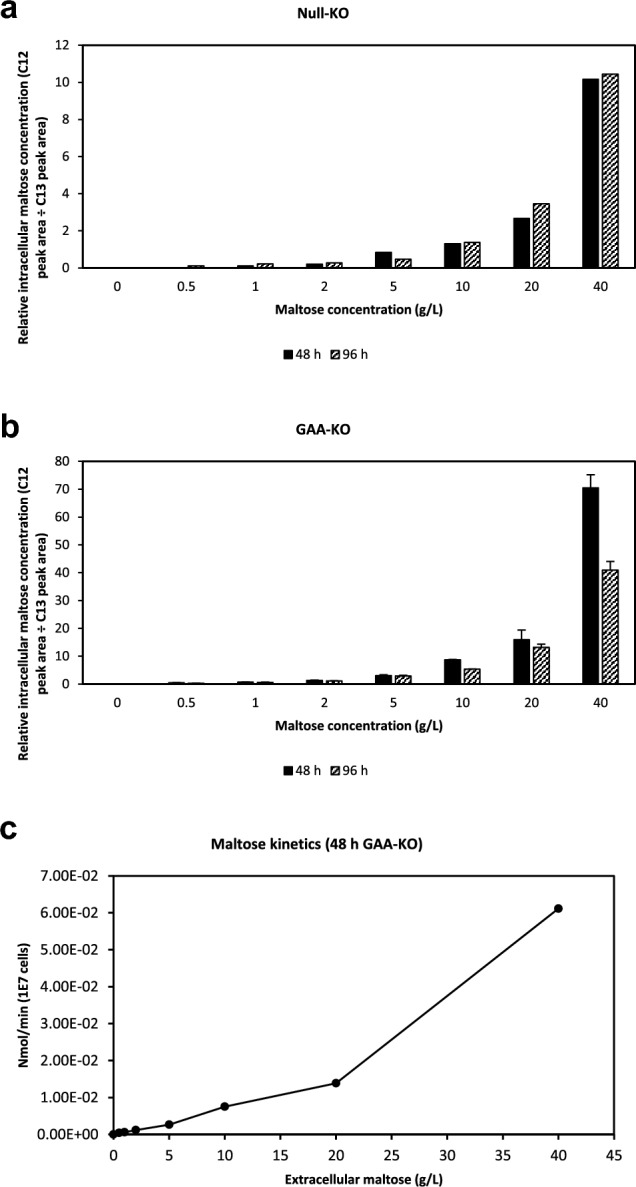
Maltose uptake kinetics in CHO-K1. GAA-KO and null-KO cells were cultivated in glucose-containing OptiCHO with maltose concentrations ranging from 0 – 40 g/L. Relative intracellular maltose concentrations were measured for (**a**) Null-KO and (**b**) GAA-KO CHO-K1 cells at 48 h (■) and 96 h (▨), and are higher for GAA-KO cells. (**c**) Maltose uptake rate in GAA-KO cells is concentration-dependent and linear up to 40 g/l. Mean and s.d values of two independent replicates plotted for the GAA-KO cell line.

GAA-KO and null-KO CHO-K1 cells were grown in 4 g/l glucose-containing OptiCHO medium supplemented with 0.5 – 40 g/L maltose, and intracellular metabolites extracted at 48 and 96 h. Culture medium osmolality increases with maltose concentration, especially at maltose concentrations exceeding 10 g/L where osmolality exceeds 320 mOsm/kg, thus exerting an inhibitory effect on growth. Intracellular maltose levels are generally higher at all maltose levels tested for GAA-KO cells, compared to null-KO cells (Fig. 7a, b). Higher extracellular maltose levels are also positively correlated with higher measured intracellular maltose, implying that maltose transport into CHO-K1 is likely to be concentration dependent. Intracellular maltose concentrations in GAA-KO cells are likely to reflect maltose uptake kinetics (Fig. 7b), while maltose concentrations in null-KO cells reflect a combination of maltose uptake and metabolism (Fig. 7a). The rate of maltose uptake for GAA-KO cells, measured per 1 × 10^7^ cells, was also observed to be linear when extracellular maltose is between 0 to 40 g/L (Fig. 7c).

## Discussion

In this study, we verified the transport of maltose into CHO-K1, and its utilization in energy metabolism. Maltose was demonstrated to be transported into CHO-K1 before being metabolized. Castanospermine, a glucosidase inhibitor that can pass through cell membranes, inhibits the growth of maltose-adapted CHO-K1, while acarbose, which cannot pass through the cell membrane, does not. GAA, an intracellular lysosomal glucosidase, was shown to be involved in intracellular maltose metabolism in the presence of glucose. After hydrolysis, labelled maltose was incorporated into various TCA cycle intermediates, confirming that maltose functions as an energy source for CHO-K1^2^. Conversely, while trehalose is able to be transported into CHO-K1 cells, it is catabolized minimally under our experimental conditions. As trehalose consists of two glucose subunits linked by an α(1 → 1)-glycosidic bond, while the glucose subunits in maltose are linked by an α(1 → 4)-glycosidic bond, this suggests that CHO-K1 cells may lack trehalase activity and hence may be unable to break down trehalose into its constituent glucose subunits. Intracellular maltose levels are higher in GAA-KO cells compared to non-KO cells, and this phenotype is restored with GAA overexpression, confirming that GAA is involved in the breakdown of maltose to glucose inside the cells. Over a 48-h period, uptake kinetics of maltose, measured in terms of nmol/min of intracellular maltose, increases linearly with extracellular maltose concentrations. Due to its size and polarity, glucose is unable to pass through the phospholipid bilayer of mammalian cell membranes via passive diffusion^16^. As maltose consists of two glucose subunits, it is thus unlikely to be able to pass through CHO-K1 cell membranes via passive diffusion. The potential CHO-K1 maltose transporter could have high V_max_ and K_m_ values, relative to extracellular maltose concentrations. Further work can be done to understand the maltose uptake mechanism into CHO-K1 and to identify potential maltose transporters.

Glucose was present in all experiments in this report, except the glucosidase inhibitor experiment. This was because CHO-K1 growth in maltose-only glucose-free medium was much slower than in glucose-containing medium, which will in turn result in long durations in cell expansion for the experiments in glucose-free medium. While glucose is present and will contribute to carbon flow into the TCA cycle, maltose metabolism is still observed using ^13^C-maltose, while minimal ^13^C was incorporated with ^13^C-trehalose. This demonstrated that maltose was metabolized in the presence of glucose. Similarly, maltose and trehalose were detected intracellularly, demonstrating that the disaccharide transport can happen in the presence of glucose. Nonetheless, the presence of glucose may have affected the observed magnitude of transport and metabolism, since competition in transport and metabolic pathways are a possibility. As most experiments were performed with two biological replicates, further work to explore maltose uptake and GAA’s role in maltose metabolism in CHO-K1 can be carried out to better understand how maltose is utilized by CHO-K1 cells.

Maltose supplementation has also been previously reported to improve CHO cell productivities while reducing lactate production and prolonging culture duration, through its combined effects as an additional carbohydrate source^2,3^ and by increasing culture medium osmolality^17^. However, there is a limit to the concentration of maltose that can be supplemented to growth media, keeping the final media osmolality between 280-320 mOsm/kg that is optimum for cell growth. Having a better understanding of maltose uptake kinetics and metabolism can allow maltose supplementation to be utilized effectively and efficiently through the development of suitable culture conditions and feeding strategies.

## Supplementary Information

Supplementary Information 1.

Supplementary Information 2.

## Data Availability

All data supporting the findings of this study are available within the paper or upon reasonable request from the corresponding author (S.K.N.).
