# Supplementary material for: Maltose metabolism in serum free CHO culture involves lysosomal acid α-glucosidase
Source: Sci Rep. 2025 Dec 4;16:1190. doi: 10.1038/s41598-025-30901-w (PMC12789618; doi:10.1038/s41598-025-30901-w)
Supplement: Supplementary file 2 — Supplementary Information 2. [file 41598_2025_30901_MOESM2_ESM.pdf]

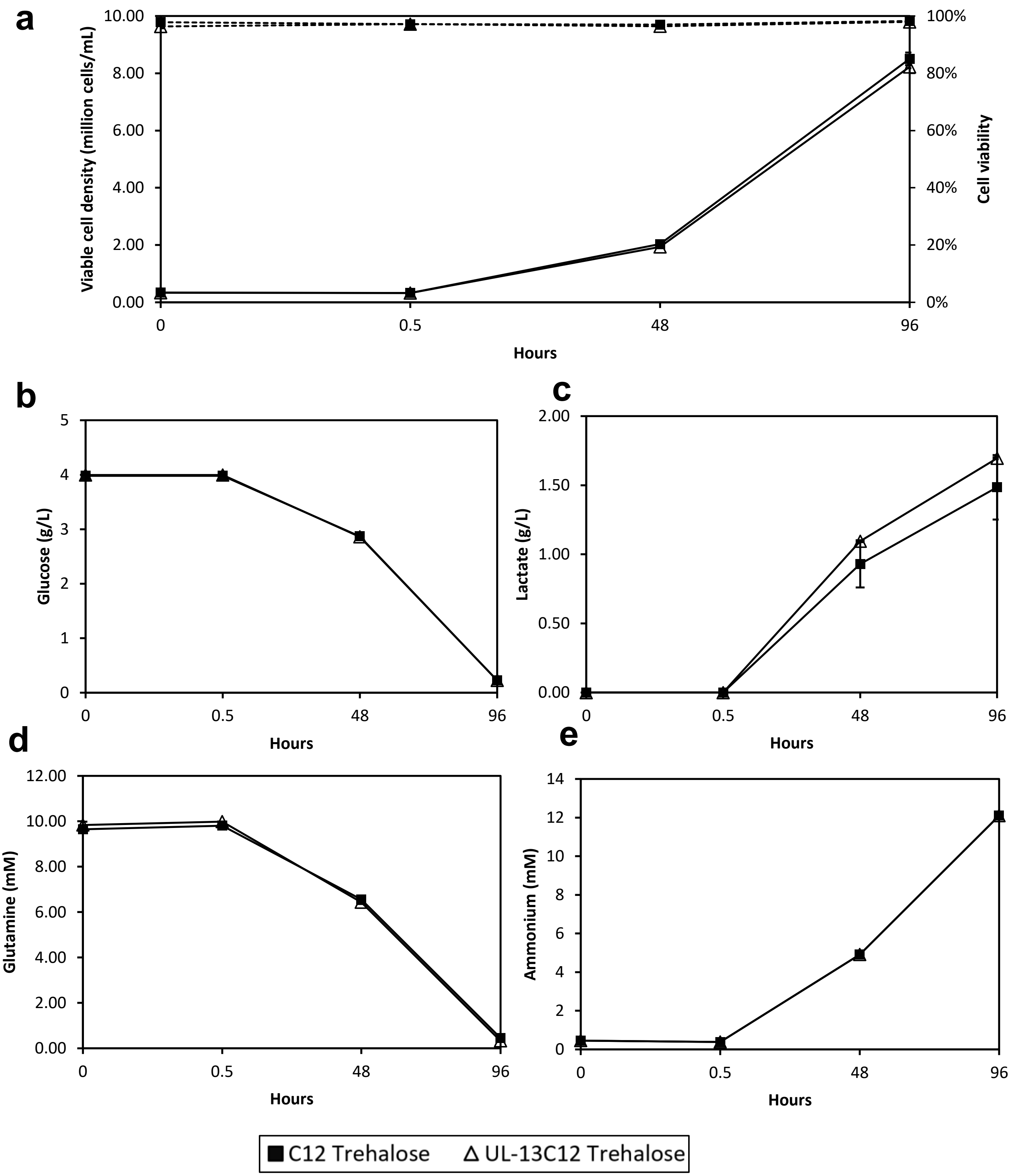

Supplementary figure S2. Growth and biochemical profiles of CHO-K1 cells cultivated in protein-free chemically defined medium (PFCDM) with natural glucose and labeled [UL-13C12] trehalose as carbohydrate sources. CHO-K1 cells originally cultured in PFCDM with 6 g/l of glucose as main carbohydrate source were sub-cultivated in PFCDM with 4 g/l glucose and either 10 g/l of natural C12 trehalose (■) or labeled [UL-13C12] trehalose (△). The cultures were monitored on at 0, 0.5, 48 and 96 h post-inoculation to obtain their (a) viable cell densities (———) and cell viabilities (┈┈┈┈) and extracellular (b) glucose, (c) lactate, (d) glutamine, (e) ammonium profiles. Mean and s.d. values from 2 independent replicates are plotted.
